# Does tailoring instructional style to a medical student’s self-perceived learning style improve performance when teaching intravenous catheter placement? A randomized controlled study

**DOI:** 10.1186/s12909-016-0720-3

**Published:** 2016-08-12

**Authors:** Dimitrios Papanagnou, Antonio Serrano, Kaitlyn Barkley, Shruti Chandra, Nicholas Governatori, Nicole Piela, Gregory K. Wanner, Richard Shin

**Affiliations:** 1 Department of Emergency Medicine, Sidney Kimmel Medical College at Thomas Jefferson University, 1020 Sansom Street, 1651 Thompson Building, Philadelphia, PA 19107 USA; 2 Sidney Kimmel Medical College, Thomas Jefferson University, 1020 Sansom Street, 1651 Thompson Building, Philadelphia, PA 19107 USA; 3 Department of Emergency Medicine, State University of New York (SUNY) Downstate Medical Center, 450 Clarkson Avenue, Brooklyn, NY 11203 USA

## Abstract

**Background:**

Students may have different learning styles. It is unclear, however, whether tailoring instructional methods for a student’s preferred learning style improves educational outcomes when teaching procedures. The authors sought to examine whether teaching to a student’s self-perceived learning style improved the acquisition of intravenous (IV) catheter placement skills. The authors hypothesized that matching a medical student’s preferred learning style with the instructor’s teaching style would increase the success of placing an IV catheter.

**Methods:**

Using the VARK model (i.e., visual [V], auditory [A], read/write [R] and kinesthetic [K]), third-year medical students reported their self-perceived learning style and were subsequently randomized to instructors who were trained to teach according to a specific learning format (i.e., visual, auditory). Success was gauged by: 1) the placement of an IV on the first attempt and 2) the number of attempts made until an IV line was successfully placed.

**Results:**

The average number of attempts in the matched learning style group was 1.53, compared to 1.64 in the unmatched learning style group; however, results were not statistically significant. Both matched and unmatched groups achieved a similar success rate (57 and 58 %, respectively). Additionally, a comparison of success between the unmatched and matched students within each learning style modality yielded no statistical significance.

**Conclusions:**

Results suggest that providing procedural instruction that is congruent with a student’s self-perceived learning style does not appear to improve outcomes when instructing students on IV catheter placement.

## Background

Over one hundred years have passed since the Flexner Report identified inadequacies in medical education and outlined a plan for reform that included standardized curricula, formation of an accreditation system, and integration of clinical skills and didactics [1, 2]. The Carnegie Foundation for the Advancement of Teaching recommended creation of curricula that individualized the learning process and increased the integration of pre-clinical and clinical content [2]. It was highlighted that medical school curricula should cultivate students’ ability to problem solve, think critically and deal with ethical dilemmas [2]. Despite these recommendations, medical education still does not provide for individualized learning and learner flexibility.

A major limitation of medical education has been its focus on the basic sciences, with modest integration of clinical skills, professionalism and ethics into the practice of clinical medicine [2, 3]. Proposed strategies for such curricular integration have included simulations, problem solving and team-based learning [3]. Integrative curricula afford the potential for improved student performance, satisfaction and quality of feedback, while simultaneously incorporating an inclusive approach to different models of learning, as described by Kolb and Gregorc [4].

The VARK model, in particular, describes four different learning styles and preferences: visual (V), auditory (A), read/write (R) and kinesthetic (K). Based on a validated inventory, the VARK model can identify student learning preferences [4]. Data surrounding the VARK model suggests that multimodal learning and teaching styles are preferred by students and result in better learning [5, 6]. Interestingly, the typical medical school curriculum does not implement an assessment to identify student learning preferences.

There is a paucity of research that investigates students’ perceived, or inventory-typed, learning style against instructors’ teaching style. To date, there is no data to support an added efficacy to matching instructors’ teaching style with students’ perceived learning style. Specifically, there is little data to suggest that matching instructors’ teaching style with students’ perceived learning style improves *procedural outcomes*. The current study examines students’ learning preferences as they apply to the instruction of peripheral intravenous (IV) catheter cannulation. The study uses elements of the VARK model to determine if there is an optimal strategy that addresses learning styles when instructing a procedural skill.

## Methods

The study enrolled a convenience sample of second-year medical students transitioning into their third year of training at [*Anonymous Medical Center*] during the month of June. All students were eligible for inclusion. Students with prior experience with IV placement were excluded from the study (i.e., students who have either observed and/or placed an IV in the past). Students could also elect not to participate in the study, while still being able to participate in the workshop. All enrolled students underwent two phases of training: 1) a didactic session on IV catheter placement; and 2) a hands-on simulation-mediated skills session on IV catheter placement.

### Didactic session

All students received a 30-min lecture on IV placement presented by a faculty member from the Department of Emergency Medicine. The goal of this basic lecture was to introduce students to intravenous placement since students are typically not immersed in the clinical setting during the first two years of medical school and, as a result, do not have the opportunity to perform procedures. This was a standard podium-style introductory lecture, using PowerPoint slides and hosted in a large auditorium that accommodated all second-year medical students. The introductory lecture took place in the morning, during a single offering, before students were randomized to practice IV placement in the afternoon. All second-year medical students attended the lecture.

Lecture content included IV indications and contraindications; a description of materials and equipment needed; and the steps to take for successful IV placement. Careful planning was given to include content delivery that considered multiple learning styles during the lecture. For this reason, slides were a mix of text content, images and video clips, which the lecturer explained in detail throughout the session. Additionally, at the start of the lecture, a standard intravenous catheter was provided to each student to better acquaint himself/herself with the device during the didactic session.

Students were then asked to complete a brief questionnaire that asked questions pertaining to demographic information (i.e., age, gender), prior history with IV placement, prior health care employment and self-perceived learning style. Because the rationale of the study focused on a *student’s self-perceived learning style*, a validated inventory was not used. Instead, students were asked to select the one learning style they felt they identify with most when learning a new procedural skill. Detailed descriptions of these learning styles were provided on the questionnaire as students made selections.

### Simulation session

Three ‘treatment arms’ were created based on the VARK model. Students were randomly assigned to one of three groups, independent of their self-perceived learning style. Groups included “V”, “A” and “K” learning-style groupings. The authors deliberately chose not to create an “R” learning-style grouping given the procedural nature of the content, as well as the time constraints for the session. Students were divided into 18 groups (6 V groups, 6 A groups and 6 K groups) and were assigned to an instructor for one hour of instructional training on IV placement. Training was spread across three hours (i.e., 6 groups trained concurrently over each hour) given limitations with space, instructors and training equipment.

Twenty faculty members from the Department of Emergency Medicine were recruited as workshop instructors and were randomly assigned to teach students according to one of the three teaching styles. Faculty instructors were trained to specifically instruct their students according to the instructor’s assigned teaching style. Instructors were provided with their own cubicle for instruction, along with a Blue Phantom™ (CAE Healthcare) low-fidelity IV task trainer with palpable veins for simulated IV placement.

Faculty instructor training consisted of a pre-briefing on the teaching strategies they could employ, as well as a checklist to help guide them during their instruction with the students. Generally, the auditory (A) instructors were advised to verbally give instructions on how to place an IV; the kinesthetic (K) instructors were advised to guide the hands of students when placing an IV; and the visual (V) instructors were advised to repeatedly demonstrate how to place an IV. All instructors had to successfully demonstrate their assigned teaching strategies to the study investigators; instructors received necessary feedback to appropriately adhere to assigned teaching styles (i.e., A, V and K) and to ensure standardization. Instructors were advised to use an additional teaching style only if: 1) the student failed to place the IV line after three attempts; or 2) instructional time for each student exceeded six minutes.

Only one student trained with a faculty instructor in a training cubicle at any given time. Students not receiving instructional training sat outside their assigned cubicle, and were not allowed to view other students practicing IV placement. Research assistants always remained in the cubicles, and recorded student outcomes and made notes on the teaching styles used by the instructors.

For each student, faculty initiated the session according to their assigned checklist. Auditory instructors provided exclusively verbal cues: they initially read [out-loud] the steps for placement; and provided verbal, just-in-time coaching to students, if needed, for each step during the procedure. Visual instructors provided minimal verbal instruction: they began the session with a brief demonstration; and, if needed, repeated demonstrations for specific steps of the procedure after students’ failed attempts. Kinesthetic instructors began the session by guiding the hands of students while they held the equipment, without actually placing the IV; if students required assistance during the procedure, instead of providing verbal cues or demonstrations, instructors would physically guide the hands of students, without taking the procedure away from them. Each student session was allotted 12 min of training time.

Successful IV cannulation for all groups was defined as return of simulated blood into the IV chamber upon piercing of the skin and threading of the IV angiocatheter through any of the task trainer’s veins. The ability to place an IV with only one attempt was considered successful. Research assistants recorded the number of attempts made by each student. After students completed the procedural workshop, they were asked to complete a brief, one-question survey that asked them to rate their instructor’s teaching style [Likert Scale: 1 (not effective) to 5 (highly effective)].

Microsoft Excel was used to analyze results. All randomly assigned groups were checked for homogeneity in terms of gender and age. Students were considered ‘matched’ if their self-perceived learning style, captured at the start of the study, matched their randomly assigned faculty instructor’s teaching style. The study was IRB approved.

## Results

**Fig. 1:**
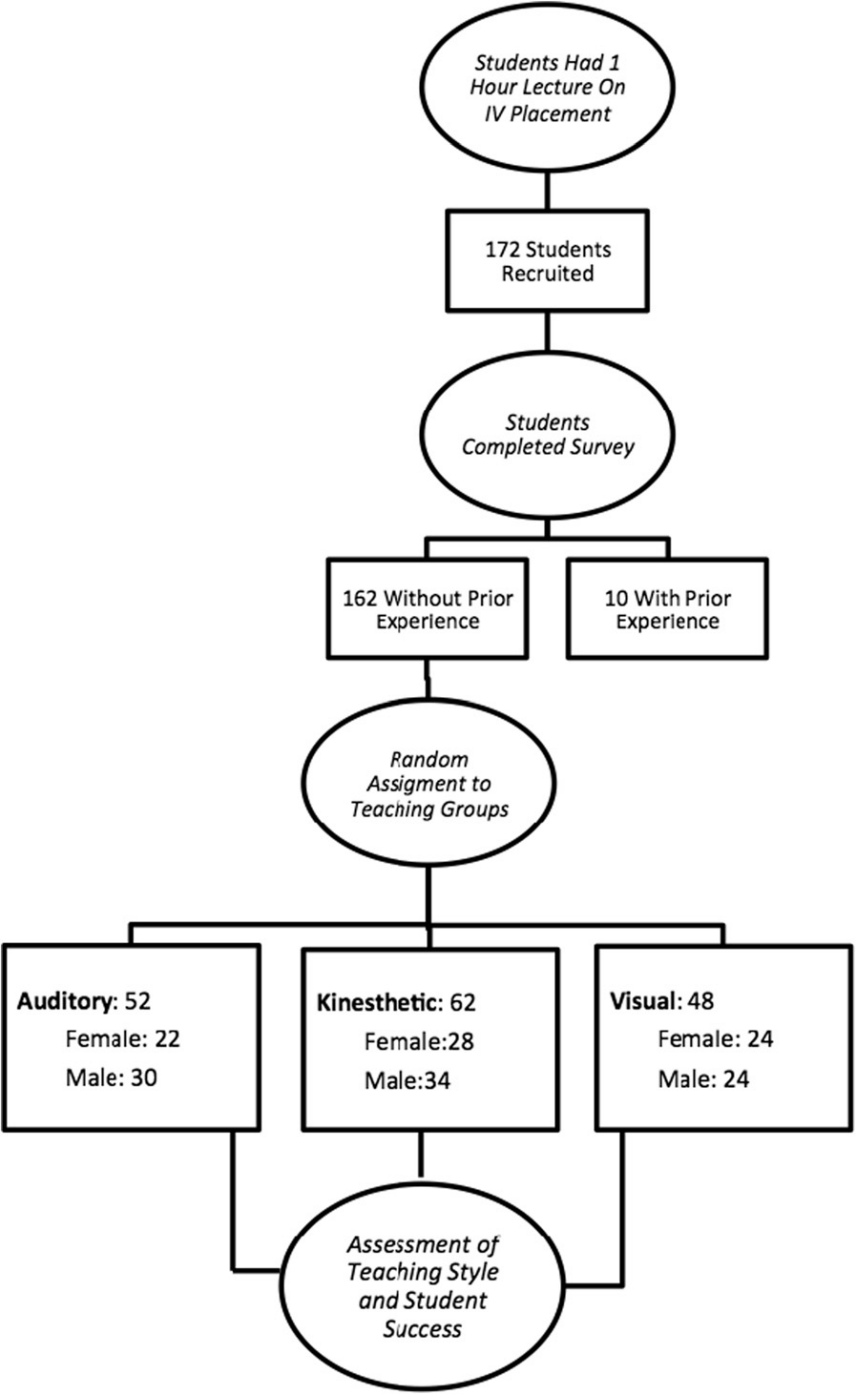
Participant assignments to instructional style ‘treatment arms’

The study aimed to enroll all 180 s-year medical students. Eight students did not provide sufficient information to be enrolled into the study. Ten additional students identified prior experience with IV placement and were excluded, leaving the final number of enrollment at 162 (Fig. 1).

Student demographic data for each group is tabulated in Table 1. The average age of students was 25 years (95 % CI, 24.6–25.8 years). Males accounted for 54 % of enrollees. Demographic data was consistent across learning-style arms. Linear regression showed no correlation between the average number of attempts and age (R^2^ = 0.0074). Gender differences for the average number of attempts were not statistically significant (*p* = 0.23).

**Table 1:** Demographics of students enrolled in study by assigned treatment groups

	Overall	Auditory	Visual	Kinesthetic
*n*	162	52	48	62
Age (yrs, avg)	25.16	24.87	25.04	25.48
Age 95 % CI	24.6-25.8	24.3-25.5	24.3-25.5	24.1-26.8
Gender (% Male)	54 %	58 %	50 %	55 %

**Fig. 2:**
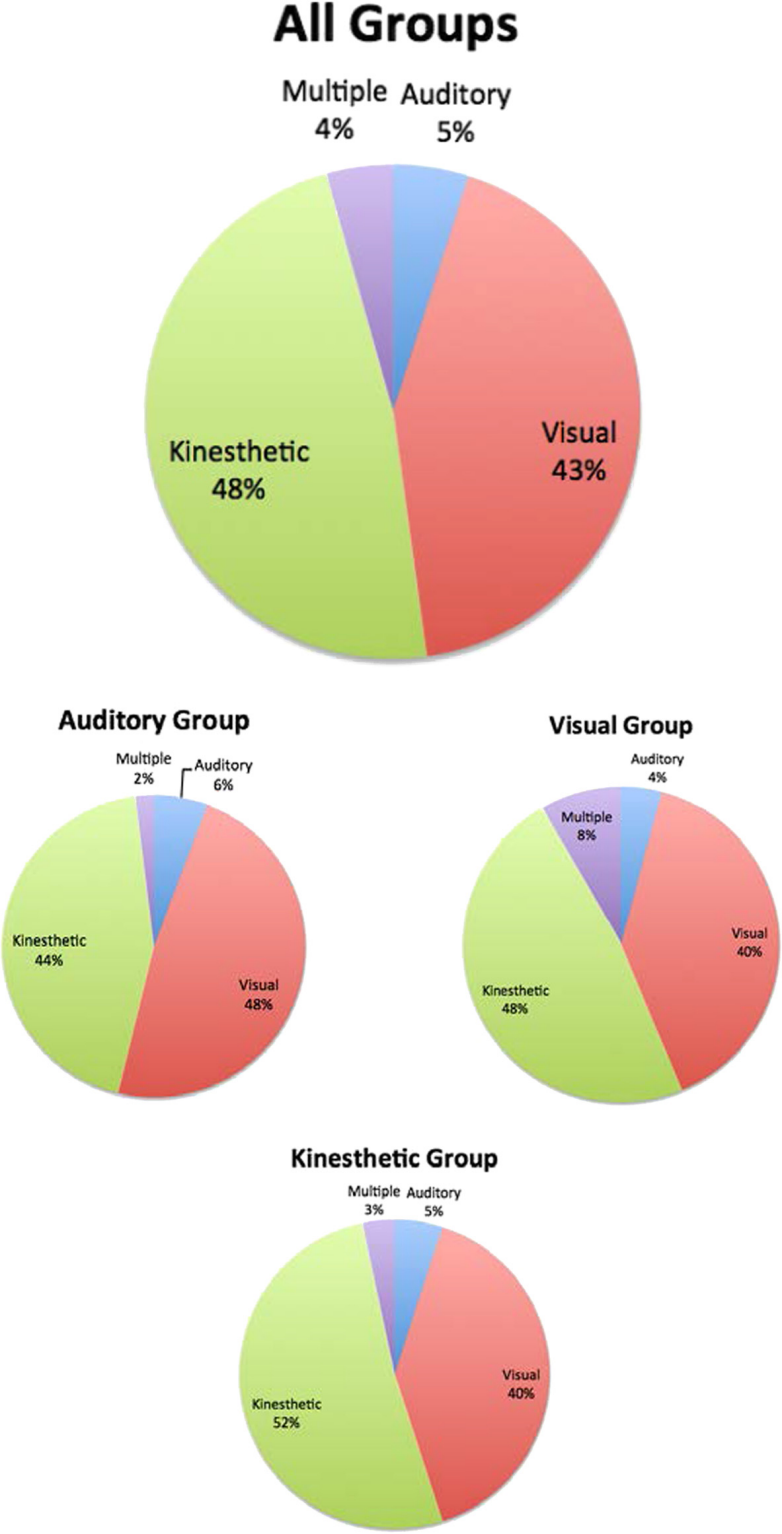
Self-reported learning style prevalence across instructional-style arms

Figure 2 shows the self-reported learning style of students. Kinesthetic learning style was most commonly reported (48 %); visual learning style was second (43 %); auditory learning style was least reported (5 %); and few students reported multiple learning modalities (4 %). Percentages of each respective learning style were similar across all assigned instructional-style arms. In each arm, the percent of self-reported visual and kinesthetic learners was the highest; visual ranged from 40–48 % and kinesthetic ranged from 44–52 %. Self-reported auditory learning style and multiple learning styles were least common and ranged from 4–6 % and 2–8 %, respectively. The differences in self-reported learning style among treatment arms were not statistically significant.

**Fig. 3:**
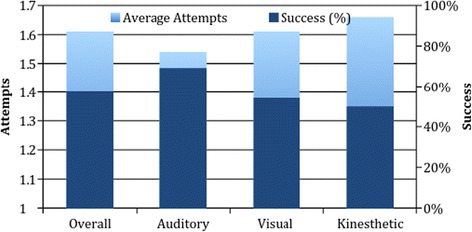
Average number of attempts and percentages of students who were successful by assigned instructional style

Figure 3 shows the average number of attempts and rates of success for students. The overall average number of attempts was 1.62. There was no statistical significance in the number of IV attempts or successful IV placement between assigned groups. Additionally, when success was examined across self-reported learning style in students, no statistical difference in the average number of attempts for IV placement was noted.

**Fig. 4:**
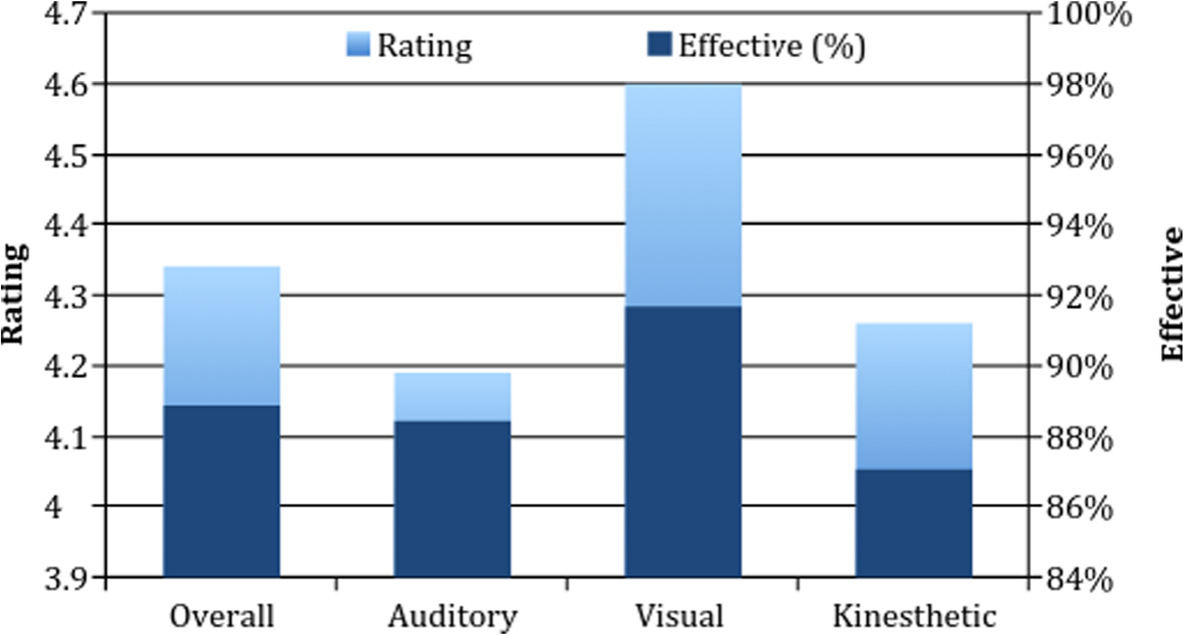
Rating of assigned instructional style and percent of students who found instructional style effective

Figure 4 shows the ratings of teaching style and the number of students who found their assigned teaching style effective. There were no statistical differences in the latter. The one statistical difference noted was in ratings of learning style: students assigned to the visual group rated this learning style higher than their counterparts in the auditory or kinesthetic groups. Likert scale ratings (1 = not effective, 5 = very effective) for the visual group (rated 4.60, CI/SD) were significantly higher than the auditory (4.19, CI/SD) or kinesthetic (4.25, CI/SD) groups (*p* < 0.05).

Table 2 shows differences between students dichotomized into matched and unmatched learning and teaching styles. The matched group had fewer average attempts; and roughly the same number of students was successful at eventual IV placement in the matched and unmatched groups. Noted findings were not statistically significant. When looking across instructional style arms, the students with a notable difference in outcomes were self-perceived kinesthetic learners who were matched to a kinesthetic instructor, when compared to their counterparts who were unmatched (χ^2^ = 3.28, *p* = 0.07). Results for matched versus unmatched learners assigned to visual and auditory groups were not statistically significant (*p* = 0.92 and *p* = 0.40, respectively). Furthermore, there was no statistical difference when comparing matched students to those with the same learning style who were unmatched (auditory, *p* = 0.47; kinesthetic, *p* = 0.77; visual, *p* = 0.91). With regards to students’ rating of teaching style, matched students, as a whole, rated the teaching style higher than their counterparts in unmatched groups (*p* = 0.07).

**Table 2:** Comparison of matched versus unmatched learning and teaching styles by assigned group

	Matched				Unmatched			
	Overall	A	V	K	Overall	A	V	K
*n*	51	3	23	25	111	49	25	37
Average attempts (#)	1.53	1.67	1.52	1.52	1.64	1.53	1.67	1.78
Success (%)	57 %	67 %	48 %	64 %	58 %	69 %	60 %	41 %
Rating	4.57	5.00	4.62	4.48	4.23	4.14	4.58	4.11

## Discussion

Consistent with previous studies, there were no statistical differences across age and gender in learning style [5, 7]. Students were least likely to self-identify with the auditory learning style, which is also consistent with prior findings [8]. Conversely, few students reported multiple learning styles, which is inconsistent with prior findings [1, 7, 9]. According to survey design and survey instructions, students were asked to choose only one learning style. Multimodal learning style was not an option, thus creating an artificial unimodal bias.

An analysis of students’ self-reported learning style against the number of attempts needed to place an IV was examined. This data could identify if students’ preference for a particular learning styles correlated with swifter success in IV placement. It appears that the self-reported kinesthetic learners needed fewer attempts to place the IV catheter, although the results did not reach significance. This may rest in the fact that the topic at task was intrinsically kinesthetic.

The authors initially hypothesized that if students’ self-perceived learning styles matched their instructors assigned teaching styles, then students would be more successful at IV placement and have fewer attempts. The data of this study does not support this hypothesis, as matching students’ learning styles with instructors’ teaching styles did not enhance IV placement.

A notable result was seen in the kinesthetic arm, where matched students were more successful than unmatched students, with success rates of 64 and 41 %, respectively (*p* = 0.07). Since these findings were not present in each treatment arm, it is possible that these findings are a result of the kinesthetic properties required to learn IV placement, paired with matched students’ preference for kinesthetic learning. Interestingly, these findings were not present when analyzing the data of other self-perceived kinesthetic learners assigned to different instructional arms. Table 2 suggests that the significance of these findings may be a result of the low success rates among unmatched students in the kinesthetic arm, more so than the higher success rates of matched students. Nonetheless, these findings suggest that matching self-reported learning style with teaching style does not significantly improve IV procedural skill acquisition with regards to both IV placement on initial attempt and number of overall attempts.

With regards to students’ perception of the teaching style used, matched students appeared to rate their assigned teaching style higher than their unmatched counterparts (4.57 out of 5, compared to 4.23 out of 5, respectively) (*p* = 0.07). When differences are analyzed by instructional arm, there is no statistical difference in student rating; although the average rating by matched students was consistently higher than unmatched students. If these findings are not due to chance, this would suggest that while matching learning style with teaching style may have little effect on procedural outcomes, matching may impact student satisfaction.

Overall, 50 % of students required the instructor to use at least two instructional styles to successfully place an IV, implying that one instructional style alone was not sufficient to achieve success. Both time constraints and repeated failure by students required instructors to use additional instructional styles. Students assigned to the kinesthetic group relied on this more, with 57 % needing an additional teaching style. Forty-nine percent of students in the visual arm required an additional instructional style, and 43 % of students in the auditory group required an additional instructional style.

The use of multiple styles to enhance student learning is consistent with findings from previous studies [1, 6]. Multimodal learning and teaching, however, was not incorporated into the study design, as the authors sought to determine only if one’s leading self-perceived learning style and the assigned instructional style influenced the effect on the number of attempts for cannulation and first-time success.

### Limitations

The study pertains to IV placement, a purely procedural skill; therefore, all findings are applicable only to procedural skill acquisition and procedural instruction. Findings cannot be generalized to non-procedural medical content. Additionally, because the current study took place during a required workshop on IV placement and was part of the students’ curriculum, uncontrollable time constraints were imposed on many aspects of the study design.

A major limitation of the study is that students’ learning style data was generated based on their self-reported preference. Students did not have the opportunity to take the VARK questionnaire. If time permitted, the authors would have gathered learning preferences based on an objective questionnaire as self-perceived learning style has the potential to be inaccurate [10]. On a similar note, the reading (R) component of the VARK was excluded, and as a result of its exclusion, study findings may have been artificially skewed towards other learning modalities, as there is no information about how many participants would have self-selected the reading learning style, had they had this opportunity. A future iteration of this study should not be limited by time constraints, and should include the reading learning style modality to prevent students from inappropriately selecting learning styles they do not readily identify with.

While the faculty instructor checklists used were not validated, the authors believe they accurately represent each instructional style modality and reflect best practices. Instructional scripts stringently adhered to VARK parameters that would most represent teaching within that instructional domain.

A larger student cohort, or perhaps a multi-centered study design, could potentially power findings of future studies. The number for enrollment was based on the size of the second-year medical student class. Prospective studies should conduct dedicated power calculations to determine if sample size is adequate enough to detect differences across matched and unmatched groups. Additionally, more powered studies should investigate the secondary teaching styles instructors have to employ when the primary teaching style is ineffective for learners, especially in light of the fact that half of the current study’s participants required more than one learning style to place an IV successfully.

The current study suggests that matching student self-perceived learning preferences to instructor teaching style does not impact procedural skill acquisition. Further investigation, however, is needed to further elucidate these findings. The study did not examine the R modality or combinations of modalities of the VARK model. Subsequent studies can incorporate not only the R modality but also multi-modal learning and instructional styles.

Furthermore, the influence of instructors’ perceived and actual learning style on student instruction, satisfaction and success can also be examined. These future modifications would help further clarify the relationship between perceived learning style, satisfaction and learning outcomes.

In the current study, success was defined as a flash of blood into the IV chamber and ability to thread the angiocatheter into the vessel on the initial attempt. The definition of success used for the study could be expanded to incorporate additional procedural aspects of IV placement (i.e., flushing the IV with saline).

Finally, secondary to time restraints of the workshop, learning outcomes were only surveyed immediately after the lecture and during the simulation of IV placement. Future studies should assess procedural retention; for example, 1 month, 6 months, and 1 year after the instructional workshop.

## Conclusion

The study concludes that matching self-perceived learning styles to instructional styles does not necessarily foster a more effective learning environment. Further investigation is needed to clarify what methods are most ideal to foster the acquisition of IV placement skills. Data suggest that incorporating multiple learning and instructional styles may be able to achieve effective learning outcomes. Determining what comprises effective procedural instruction, however, is required to better foster learning, retention and student satisfaction.
